# Altered Inter‐Network Functional Integration Underlies Speech Impairment in Parkinson's Disease: A Resting‐State fMRI Analysis

**DOI:** 10.1002/brb3.71798

**Published:** 2026-09-28

**Authors:** Shan Deng, Yongqing Xiao, Zimei Dong, Cai Zhong, Yu Yu, Chunxiao Yang, Liya Pan, Baohui Weng, Yuan Chen, Ziming Ye, Ying Liu, Chao Qin

**Affiliations:** ^1^ Department of Neurology Liuzhou Workers' Hospital Liuzhou Guangxi China; ^2^ Department of Neurology The First Affiliated Hospital of Guangxi Medical University Nanning Guangxi China; ^3^ Department of Neurology People's Hospital of Chuxiong Yi Autonomous Prefecture Chuxiong Yunnan China; ^4^ Department of Neurology Minzu Hospital of Guangxi Zhuang Autonomous Region Nanning Guangxi China; ^5^ Department of Neurology People's Hospital of Guangxi Zhuang Autonomous Region Nanning Guangxi China; ^6^ Department of Neurology The Second Affiliated Hospital of Guangxi Medical University Nanning Guangxi China

**Keywords:** functional network connectivity, independent component analysis, Parkinson's disease, rs‐fMRI, speech disorders, speech impairment

## Abstract

**Background:**

Speech impairment is common in Parkinson's disease (PD) and lacks effective clinical interventions due to poorly understood underlying mechanisms. Therefore, this study aimed to explore the relationship between altered brain functional networks and speech impairments in PD, identifying neural correlates of abnormal brain activity linked to PD‐related speech disorders (SD).

**Methods:**

Baseline clinical characteristics, neurobehavioral assessments, and resting‐state functional magnetic resonance imaging (rs‐fMRI) data were collected from three groups: PD individuals with SD (PD‐SD group), PD individuals without SD (PD‐NON‐SD group), and healthy controls (HC group). Independent component analysis (ICA) was performed to identify resting‐state networks (RSNs) using the Group ICA of fMRI Toolbox (GIFT) implemented in MATLAB. Functional network connectivity (FNC) was subsequently quantified by calculating Pearson correlation coefficients between the extracted RSNs. One‐way analysis of variance (ANOVA) was used to assess group differences in FNC, followed by post hoc pairwise comparisons for networks showing significant effects. Given the reported association between speech impairment and cognitive impairment in PD, correlation analyses were further performed to investigate the relationships between FNC alterations and clinical measures, including speech performance and cognitive function assessed by the Montreal Cognitive Assessment (MoCA).

**Results:**

Significant differences in FNC were observed between the left frontoparietal network (LFPN) and auditory network (AUN) across the three groups (*p* = 0.0280). In particular, the FNC between LFPN and AUN was significantly stronger in the PD‐SD group compared to the PD‐NON‐SD group (*p* = 0.0221) and the HC group (*p* = 0.0047). Spearman correlation analysis revealed significant positive correlation between LFPN‐AUN functional connectivity and Unified Parkinson's Disease Rating Scale part III (UPDRS‐III) speech subscores (*r*_s_ = 0.2624, *p* = 0.0466), as well as a significant negative correlation with MoCA scores (*r*_s_ = −0.2847, *p* = 0.0249).

**Conclusion:**

This study identifies disrupted functional connectivity between the LFPN and AUN in individuals with PD‐SD. These findings suggest that altered integration of RSNs involved in motor, cognitive, and auditory processing may contribute to speech impairment in PD. Hyperconnectivity between LFPN and AUN may thus serve as a neural substrate for speech impairment in PD. Specifically, increased connectivity between the LFPN and AUN may represent a neural signature associated with speech dysfunction in PD. These results provide potential mechanistic insights and highlight candidate neural targets for future neuromodulation strategies aimed at improving speech function.

## Graphical abstract

Resting‐state functional magnetic resonance imaging and independent component analysis were used to compare large‐scale functional network connectivity among patients with Parkinson's disease with speech disorders (PD‐SD, *n* = 62), patients without speech disorders (PD‐NON‐SD, *n* = 16), and healthy controls (HC, *n* = 36). Patients with PD‐SD exhibited significantly increased connectivity between the left frontoparietal network (LFPN) and auditory network (AUN) compared with both PD‐NON‐SD participants and healthy controls. Greater LFPN–AUN connectivity was associated with more severe speech impairment and poorer cognitive performance in unadjusted correlation analyses. These findings suggest that maladaptive integration between cognitive‐control and auditory‐processing networks may contribute to speech impairment in Parkinson's disease and may represent a potential target for future neuromodulation.

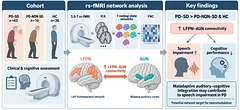

## 1 Introduction

Parkinson's disease (PD) is a progressive neurodegenerative disorder that predominantly affects older adults, with epidemiological studies consistently demonstrating a higher prevalence among individuals aged ≥ 60 years (Elbaz et al. 2016; Qi et al. 2021; Li et al. 2019). However, early‐onset Parkinson's disease (EOPD) has emerged as an increasingly recognized clinical subgroup, with recent reports from the International Parkinson and Movement Disorder Society emphasizing the importance of younger individuals within the broader PD spectrum (Andrews et al. 2024). The neuropathological hallmarks of PD include progressive degeneration of dopaminergic neurons in the substantia nigra and the accumulation of Lewy bodies. These pathological changes gradually extend to subcortical and cortical regions, resulting in a heterogeneous clinical phenotype characterized by both motor and non‐motor manifestations (Qi et al. 2021; Tanner and Ostrem 2024; Braak et al. 2003).

Speech disorders (SD) represent one of the most prevalent and clinically significant non‐motor symptoms of PD, affecting more than 90% of individuals with the disease (Harel et al. 2004). These deficits may occur early in the disease progression and can occasionally precede the onset of motor symptoms. The clinical manifestations are highly heterogeneous and include reduced vocal loudness (hypophonia), impaired prosody (such as reduced stress and monopitch), imprecise articulation (including consonantal slurring), altered speech rate (including short rushes, inappropriate pausing, and variable speed), and altered voice quality (such as breathiness, hoarseness, and harshness) (Moreau and Pinto 2019). These impairments substantially compromise communication ability, social engagement, and overall quality of life (Diao et al. 2024). Moreover, disease‐related alterations in speech and voice characteristics may serve as potential early behavioral biomarkers for PD diagnosis (Hlavnička et al. 2017; Rusz et al. 2022; Kowalska‐Taczanowska et al. 2020).

Current models suggest that PD‐related speech impairment arises from multiple interacting mechanisms, including motor dysfunction caused by basal ganglia circuit abnormalities (e.g., reduced hypokinetic drive), disrupted sensorimotor integration involving impaired auditory feedback processing (Ho et al. 2000; H. Liu et al. 2012; Arnold et al. 2014), cognitive deficits affecting attention and working memory (Y. Liu et al. 2019), and altered functional connectivity between the subthalamic nucleus and speech‐related neural networks (Cai et al. 2024). Recent evidence indicates that normal speech production depends critically on sensorimotor control mechanisms that integrate auditory feedback with motor commands. Consistent with this framework, individuals with PD exhibit impaired auditory‐vocal integration, reflecting disrupted coordination between sensory processing and motor execution during speech production (Dai, Chen, et al. 2022; Dai, Wang, et al. 2022). Specifically, dopamine depletion within the striatum reduces the automaticity of speech‐related motor control, increasing reliance on higher‐order cognitive processes, including working memory and attention, to consciously plan, sequence, and monitor articulatory movements. Consequently, deterioration of these compensatory cognitive resources may further aggravate speech dysfunction in PD (Wu et al. 2015).

In this study, we used resting‐state functional magnetic resonance imaging (rs‐fMRI) combined with independent component analysis (ICA) to investigate alterations in functional connectivity among large‐scale resting‐state networks (RSNs) in PD individuals with SD (PD‐SD group), PD individuals without SD (PD‐NON‐SD group) and healthy controls (HC group). We further examined the associations between aberrant functional network connectivity (FNC) patterns and clinical characteristics, with a particular emphasis on speech and cognitive performance. This study aimed to elucidate the neural mechanisms underlying PD‐related speech impairment by characterizing the interactions among motor, auditory–motor integration, and cognitive systems, thereby providing insights into potential neural targets for future neuromodulation strategies.

## 2 Methods

### 2.1 Subjects

A total of 78 individuals with PD and 36 HCs were enrolled in this study. Among the PD cohort, 62 individuals presented with SD. All participants with PD were diagnosed according to the clinical diagnostic criteria established by the UK Parkinson's Disease Society Brain Bank, based on medical history and clinical manifestations (Postuma et al. 2015). Participants were aged 30–85 years, with no sex restrictions, and had Hoehn and Yahr (H&Y) stages ranging from 1 to 5. Initial clinical assessments were performed by a senior neurologist specializing in movement disorders with extensive experience in administering the Movement Disorder Society–Unified Parkinson's Disease Rating Scale (MDS‐UPDRS). Speech impairment assessments were independently reviewed and confirmed by an otolaryngology specialist with expertise in voice disorders. Specifically, individuals with PD and SD were required to have an MDS‐UPDRS part III speech item score ≥ 1, indicating at least mild impairment in speech expression (Y. Liu et al. 2019). In contrast, individuals with PD without SD were required to have an MDS‐UPDRS part III speech item score of 0 (Y. Liu et al. 2019). Exclusion criteria included a history of psychoactive substance or alcohol abuse; other organic central nervous system disorders, including stroke, vascular cognitive impairment, Alzheimer's disease, dementia with Lewy bodies, multiple system atrophy, severe white matter disease, and epilepsy; severe neurological deficits, such as visual or auditory impairment; severe systemic diseases with an expected survival time of < 90 days; psychiatric disorders, including bipolar disorder and schizophrenia; contraindications to magnetic resonance imaging (MRI); left‐handedness; and inability to comply with the study protocol. In addition, 36 HCs matched for age, sex, linguistic background, and educational level were recruited. HCs had no history of significant medical or psychiatric disorders or substance abuse and achieved a Montreal Cognitive Assessment (MoCA) score ≥ 25, which represents an optimal education‐adjusted cutoff for defining normal cognition in the Chinese population (Lu et al. 2011).

Written informed consent was obtained from all participants or their legally authorized representatives prior to study enrollment. The study protocol was approved by the Ethics Committee of Liuzhou Workers’ Hospital (Approval No. KY2021024).

### 2.2 Clinical Assessments

Disease severity and clinical stage were evaluated in all 78 individuals with PD using the MDS‐UPDRS and H&Y scales. Specifically, the complete MDS‐UPDRS part III motor examination was administered to all participants with PD, and the speech item score was used to assess speech expression and verbal function. Cognitive performance was evaluated using the MoCA and Mini‐Mental State Examination (MMSE), whereas emotional status was assessed using the Hamilton Anxiety Rating Scale (HAMA) and Hamilton Depression Rating Scale (HAMD). Participants receiving antiparkinsonian medication underwent all neurobehavioral assessments and rs‐fMRI scanning during the ON‐medication state. Medication dosages were converted into levodopa equivalent daily doses (LEDDs) according to a standard conversion formula (Tomlinson et al. 2010). The overall study workflow is illustrated in Figure 1.

**FIGURE 1:**
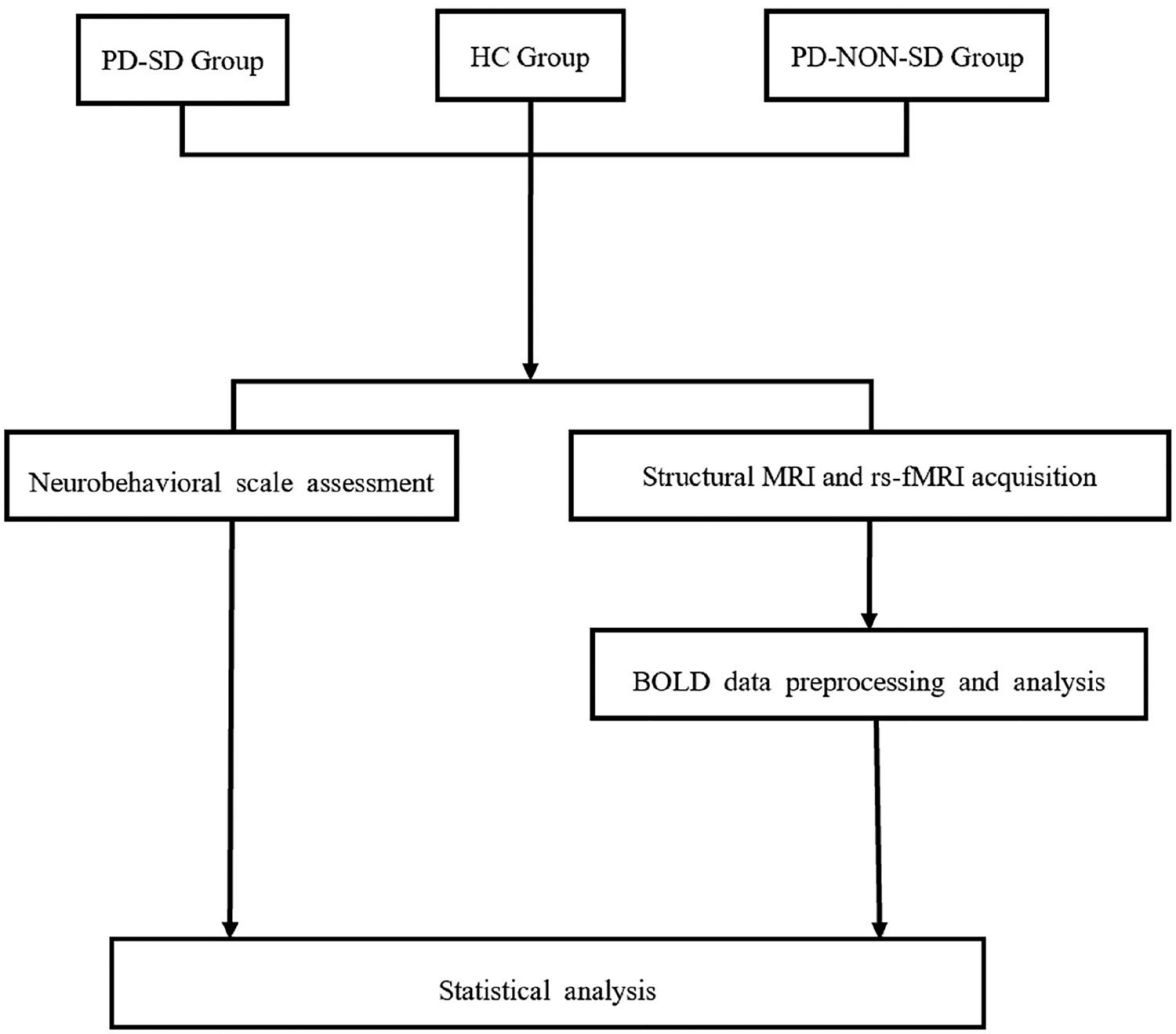
Overview of the study workflow.

### 2.3 MRI Acquisition

Blood oxygen level‐dependent (BOLD) data were acquired using a 32‐channel head coil on a Philips Ingenia 3.0T CX MRI scanner. rs‐fMRI was performed with a gradient echo imaging sequence using the following parameters: TR = 2000 ms; TE = 30 ms; flip angle = 90°; scan field = 240 × 240 mm^2^; matrix = 80 × 80; slice thickness = 4 mm; slice spacing = 0 mm; 88 time points with 40 slices per time point. High‐resolution 3D T1‐weighted structural images were acquired with a gradient echo imaging sequence using the following parameters: TR = 6.6 ms; TE = 3.0 ms; scan field = 240 × 240 × 180 mm; voxel size = 1 × 1 × 1 mm; matrix = 240 × 240; slice thickness = 1 mm. Participants were instructed to lie supine with their eyes closed during scanning and wore earplugs to reduce noise disturbance.

### 2.4 Image Post‐Processing

rs‐fMRI data preprocessing was performed using SPM12 and REST+ tools on the MATLAB platform, following these steps: format conversion, removal of the first 10 time points, slice timing correction, head motion correction, spatial normalization to MNI space (resampled to 3 mm^3^ voxels), spatial smoothing (full‐width at half maximum = 6 mm), linear drift removal, regression of covariates (e.g., white matter, cerebrospinal fluid, and 24 head motion parameters), and bandpass filtering (0.01–0.08 Hz). The preprocessed data were then subjected to ICA and FNC analysis.

### 2.5 Analysis of Functional Connectivity in Large‐Scale RSNs Using ICA

Post‐processing involved performing ICA on the preprocessed rs‐fMRI data using the GIFT software package (version 4.0.3.5) on the MATLAB platform (version 2022b). The analysis included the following steps: (1) ICA decomposition: group‐level ICA was conducted with the number of components set to 30; (2) component stability assessment: the ICASSO toolkit (repeated 20 times) was used to ensure stability of the decomposition results; (3) brain network identification: identified spatial independent components (ICs) were matched with known RSN templates and anatomical knowledge to extract relevant RSNs; and (4) FNC calculation: Pearson correlation coefficients between the time series of each RSN were calculated for each group, followed by Fisher's *z*‐transformation to obtain *z*‐values representing the FNC strength between RSNs.

### 2.6 Statistical Analysis

Statistical analysis was performed using SPSS 25.0 software. Data normality was assessed before analysis. For normally distributed continuous data, results were expressed as mean ± standard deviation. Comparisons between two groups were made using independent samples *t*‐test, while one‐way analysis of variance (ANOVA) was used for comparisons among three groups. Post hoc pairwise comparisons were conducted using the LSD‐t test. Non‐normally distributed data were expressed as median (interquartile range), with group comparisons performed using the Mann–Whitney *U* test (two groups) or the Kruskal–Wallis *H* test (three groups), followed by Bonferroni correction for multiple comparisons. For categorical data, chi‐square tests were applied. Pearson correlation analysis was performed on the RSN components extracted by ICA, followed by Fisher's *z*‐transformation. FNC analysis was conducted within the GIFT software package and corrected for false discovery rate (FDR). ANOVA was used to compare FNC values among the three groups, and significant differences were further evaluated using post hoc tests with the Bonferroni correction for multiple comparisons. Partial correlation analysis was conducted between FNC values and clinical indicators in PD‐SD individuals, with statistical significance set at *p* < 0.05.

## 3 Results

### 3.1 Demographic Data and Clinical Characteristics

A total of 62, 16, and 36 individuals were included in the PD‐SD, PD‐NON‐SD, and HC groups, respectively. As shown in Table 1, no significant differences were observed among the three groups in terms of sex, age, or educational level (*p* > 0.05). Compared with the HC group, both PD groups exhibited significantly higher HAMD and HAMA scores, as well as lower MMSE and MoCA scores (*p* < 0.05). No significant differences were detected between the PD‐SD and PD‐NON‐SD groups in disease duration or LEDD. However, individuals in the PD‐SD group demonstrated higher MDS‐UPDRS part III total scores, more advanced H&Y stages, and lower MoCA scores than those in the PD‐NON‐SD group (*p* < 0.05).

**TABLE 1:** Demographic characteristics and clinical data of the PD‐SD, PD‐NON‐SD, and HC groups.

Characteristics	PD‐SD group (*n* = 62)	PD‐NON‐SD group (*n* = 16)	HC group (*n* = 36)	*p*‐value
Gender (female/male)	35/27	8/8	15/21	0.3680
Age (years)	65.63 ± 6.63	63.88 ± 8.38	62.5 ± 7.5	0.1590
Education level	7.94 ± 2.19	8.63 ± 3.38	7.50 ± 1.50	0.5800
Duration of illness (years)	3.50 ± 2.50	3.50 ± 2.50	NA	0.4950
H&Y stage	2.50 ± 0.50	2.00	NA	0.0020^b^
LEDD (mg)	275.00 ± 275.00	37.50 ± 37.50	NA	0.1000
MMSE	23.38 ± 3.63	27.00 ± 1.75	29.00 ± 1.00	< 0.0001^a^ 0.1730^b^
MoCA	16.13 ± 5.13	23.00 ± 2.00	25.50 ± 0.50	< 0.0001^a^ 0.0180^b^
UPDRS‐III	34.76 ± 11.00	19.63 ± 9.13	NA	< 0.0001^b^
HAMA	14.00 ± 5.00	8.00 ± 2.00	6.50 ± 1.50	< 0.0001^a^ 0.0970^b^
HAMD	13.00 ± 4.25	10.00 ± 3.00	7.00 ± 1.00	< 0.0001^a^ 0.6450^b^

Abbreviations: H&Y, Hoehn and Yahr; HAMA, Hamilton Anxiety Rating Scale; HAMD, Hamilton Depression Rating Scale; HC, healthy control; LEDD, levodopa equivalent daily dose; MMSE, Mini‐Mental State Examination; MoCA, Montreal Cognitive Assessment; NA, not applicable; PD, Parkinson's disease; SD, speech disorders; UPDRS‐III, Unified Parkinson's Disease Rating Scale part III.

^a^ Differences observed between PD‐SD, PD‐NON‐SD, and HC groups.

^b^ *p*‐value for comparison between PD‐SD and PD‐NON‐SD groups.

### 3.2 RSN Functional Connectivity Changes

Using ICA combined with neuroanatomical characterization, we identified seven RSNs across all participants. As shown in Table 2 and Figure 2, these networks comprised the left frontoparietal network (LFPN), visual network, auditory network (AUN), anterior default mode network (aDMN), salience network (SN), posterior default mode network (pDMN), and right frontoparietal network (RFPN). Notably, the AUN identified in this study encompassed key auditory and multisensory integration regions, including the superior temporal gyrus, transverse temporal gyrus (primary and secondary auditory cortices), temporal pole, and insula. This spatial distribution was highly consistent with established large‐scale RSN templates (Beckmann et al. 2005; Smith et al. 2009). Functionally, the AUN represents a core neural system involved in auditory processing and sensorimotor feedback integration.

**TABLE 2:** Resting‐state networks identified in PD‐SD, PD‐NON‐SD, and HC groups using independent component analysis.

Functional network connectivity	MNI peak coordinates			Core brain regions
	*X*	*Y*	*Z*	
Left frontoparietal network	−50.5	20.5	−0.5	Left middle frontal gyrus, left inferior frontal gyrus, left superior frontal gyrus, left inferior parietal lobule, left angular gyrus, left superior parietal gyrus, and left precuneus.
Visual network	0.5	−78.5	29.5	Occipital visual cortex
Auditory network	−42.5	−15.5	11.5	Bilateral superior temporal gyrus, transverse temporal gyrus, temporal pole, and lateral and posterior insula of the superior temporal gyrus.
Anterior default mode network	0.5	56.5	3.5	Medial prefrontal cortex and medial temporal lobe.
Salience network	−44.5	−2.5	−2.5	Bilateral insula, medial frontal gyrus, superior frontal gyrus, infraorbital gyrus, anterior cingulate cortex, and supplementary motor area.
Posterior default mode network	0.5	−51.5	23.5	Cuneus, angular gyrus, and posterior cingulate cortex.
Right frontoparietal network	44.5	−53.5	47.5	Right middle frontal gyrus, right superior frontal gyrus, right inferior parietal lobule, right angular gyrus, right superior parietal gyrus, and right superiormarginal gyrus.

Abbreviations: HC, healthy control; PD, Parkinson's disease; SD, speech disorders.

**FIGURE 2:**
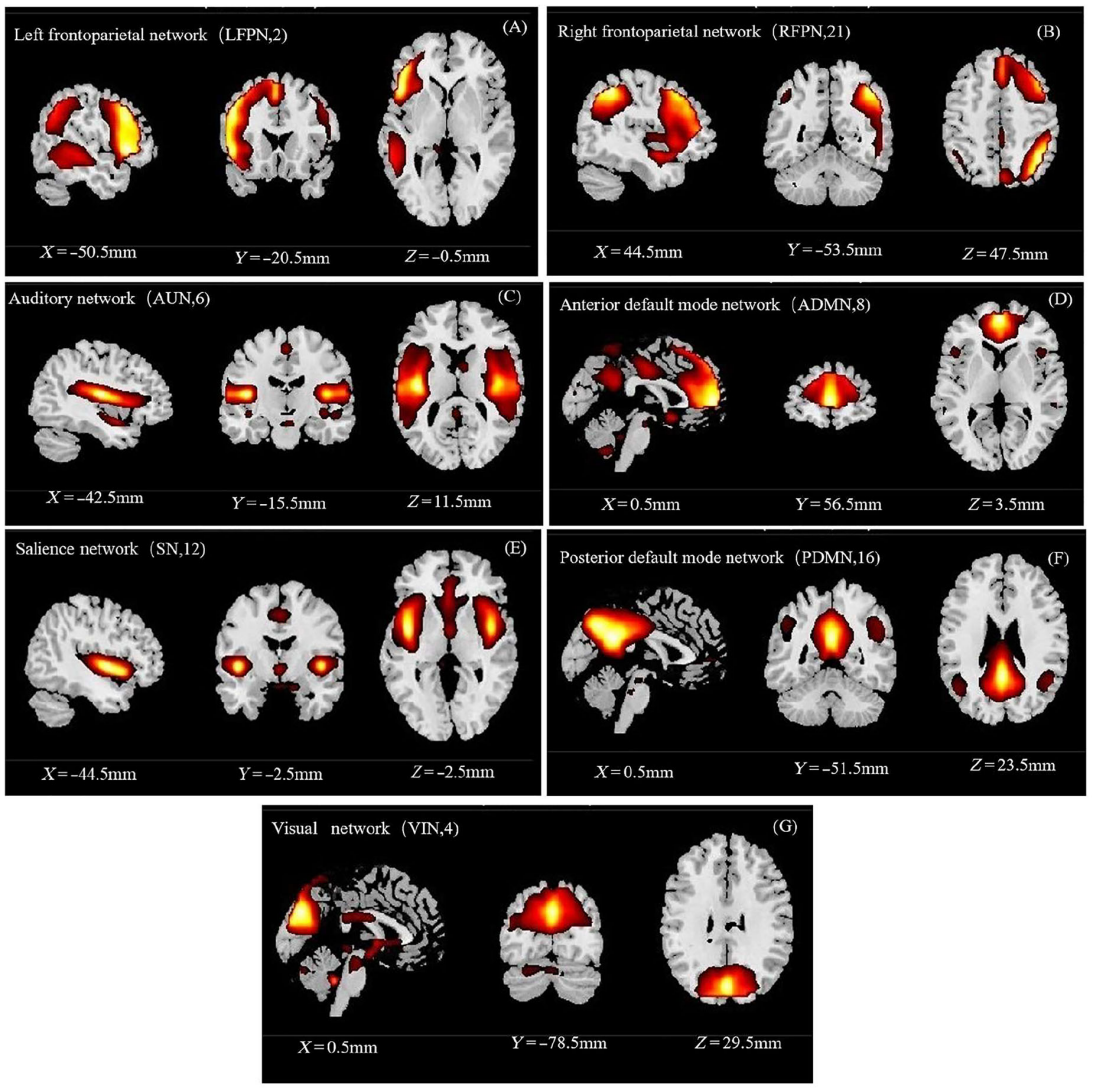
Resting‐state networks identified by ICA in the PD‐SD, PD‐NON‐SD, and HC groups. (A) Left frontoparietal network; (B) right frontoparietal network; (C) auditory network; (D) anterior default mode network; (E) salience network; (F) posterior default mode network; and (G) visual network.

Group‐averaged static functional connectivity (sFC) between pairs of ICs was calculated using the BOLD signals from the three groups. The seven ICA‐derived components and their corresponding RSNs were reordered according to network classification, as illustrated in Figure 3. Figure 3 presents group‐level functional connectivity matrices at both the components and network levels, respectively. The matrix values represent Fisher's *z*‐transformed Pearson correlation coefficients.

**FIGURE 3:**
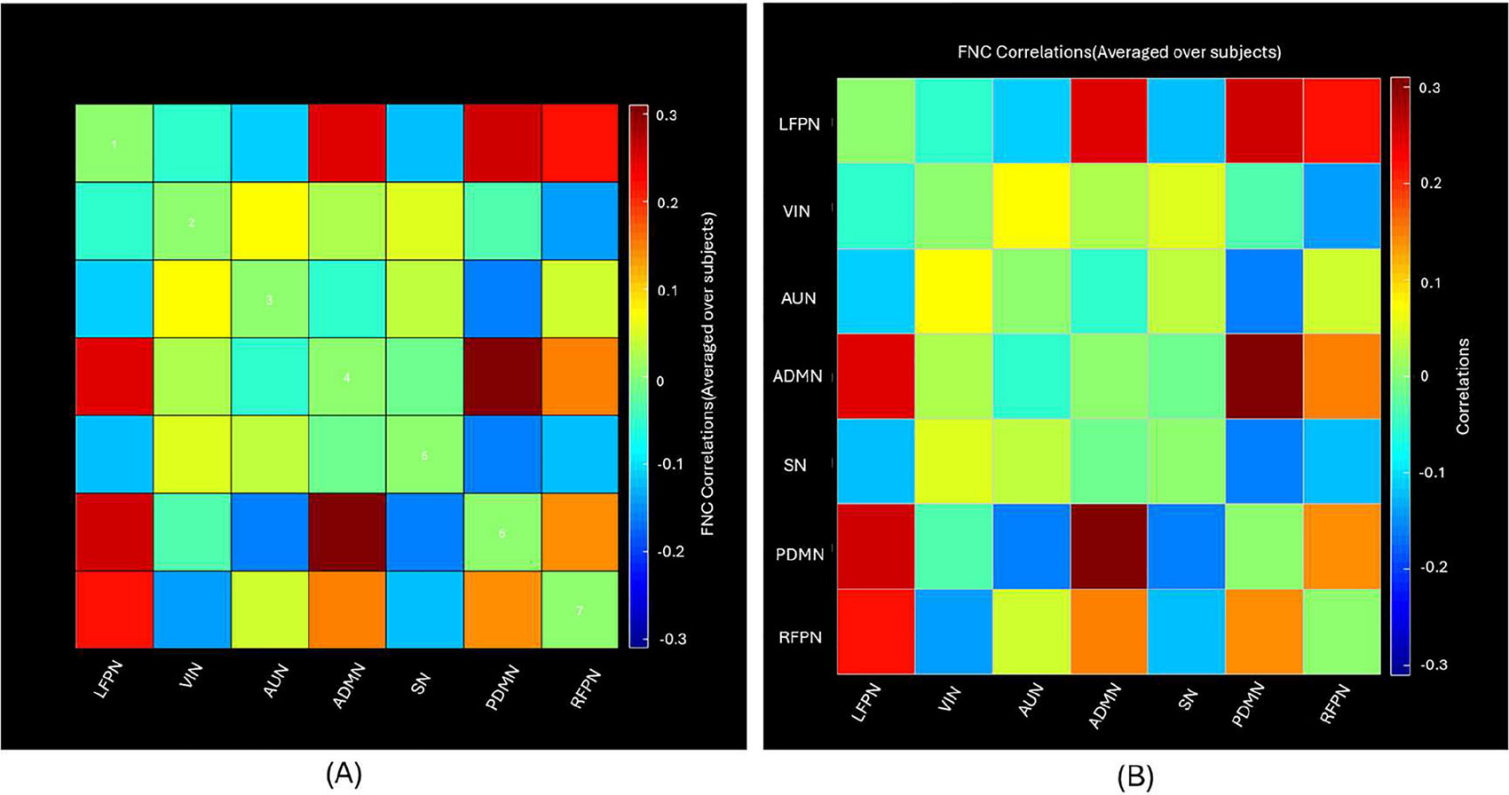
Functional connectivity matrices derived from ICA components and resting‐state networks. (A) Functional connectivity matrix among the seven independent components identified by ICA in the PD‐SD, PD‐NON‐SD, and HC groups. (B) Functional connectivity matrix among the seven resting‐state networks identified by ICA in the PD‐SD, PD‐NON‐SD, and HC groups. HC, healthy control; ICA, independent component analysis; PD, Parkinson's disease; SD, speech disorders.

Significant group differences in FNC were observed between the LFPN and AUN across the PD‐SD, PD‐NON‐SD, and HC groups (*p* = 0.0280, FDR‐corrected). Post hoc analyses revealed that LFPN–AUN connectivity was significantly increased in the PD‐SD group compared with both the PD‐NON‐SD group (*p* = 0.0221) and the HC group (*p* = 0.0047), whereas no significant difference was detected between the PD‐NON‐SD and HC groups (*p* > 0.9999). The FNC matrices and between‐group comparisons are presented in Figure 4.

**FIGURE 4:**
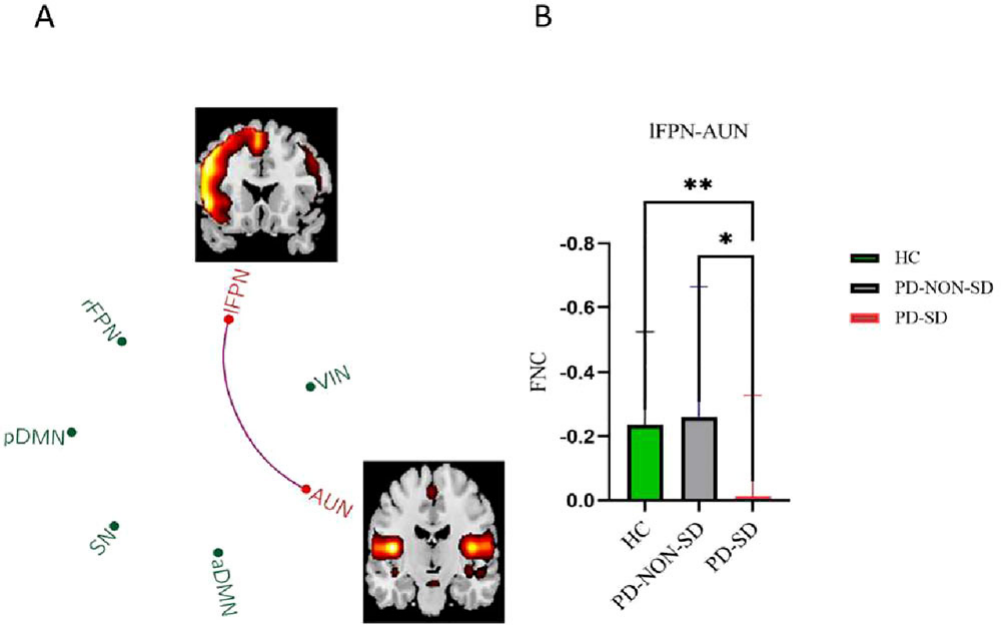
Abnormal functional connectivity between the left frontoparietal network and auditory network in the PD‐SD, PD‐NON‐SD, and HC groups, with pairwise comparisons of functional network connectivity values. An omnibus one‐way ANOVA with FDR correction was first performed across the entire functional connectivity matrix, identifying the LFPN–AUN connection as significantly different among the three groups (*p* = 0.0280). Subsequent post hoc Bonferroni‐corrected comparisons revealed group‐specific differences. (A) Schematic representation of the spatial maps of the LFPN and AUN analyzed in this study. (B) Bar graph showing post hoc pairwise comparisons of FNC values among groups. *p < 0.05; **p < 0.01. Error bars represent standard deviations. ANOVA, analysis of variance; AUN, auditory network; FDR, false discovery rate; FNC, functional network connectivity; HC, healthy control; LFPN, left frontoparietal network; PD, Parkinson's disease; SD, speech disorders.

### 3.3 Correlation Between Inter‐Network FNC and Clinical Metrics

To investigate the associations between altered FNC and clinical manifestations in individuals with PD‐SD, partial correlation analyses were performed. As shown in Table 3, no significant correlations were observed between LFPN–AUN FNC values and any of the assessed clinical parameters.

**TABLE 3:** Correlation analysis between LFPN‐AUN FNC values and clinical indicators in the PD‐SD group.

Clinical indicators	Left frontoparietal network—auditory network FNC value	
	*r*	*p*
Age	0.0540	0.7170
Education level	−0.2850	0.0500
Duration of illness	−0.1730	0.2390
H&Y stage	−0.0710	0.6330
LEDD	0.0230	0.8760
UPDRS‐III total score	0.1190	0.4220
MMSE	0.0520	0.7260
MOCA	−0.0160	0.9140
HAMA	0.0450	0.7600
HAMD	−0.0400	0.7860

*Note*: r‐values represent correlation coefficients derived from partial correlation analysis, controlling for age, gender, and disease duration. Statistical significance was set at *p* < 0.05.

Abbreviations: AUN, auditory network; FNC, functional network connectivity; H&Y, Hoehn and Yahr; HAMA, Hamilton Anxiety Rating Scale; HAMD, Hamilton Depression Rating Scale; LEDD, levodopa equivalent daily dose; LFPN, left frontoparietal network; MMSE, Mini‐Mental State Examination; MoCA, Montreal Cognitive Assessment; PD, Parkinson's disease; SD, speech disorders; UPDRS‐III, Unified Parkinson's Disease Rating Scale part III.

### 3.4 Correlation Between LFPN–AUN Functional Connectivity and Clinical Indicators

We further investigated the associations between LFPN–AUN functional connectivity and clinical indicators in individuals with PD‐SD (Figure 5). Before adjustment for potential confounding factors, Spearman correlation analysis revealed a significant positive correlation between the LFPN–AUN functional connectivity and MDS‐UPDRS part III speech item scores (*r*_s_ = 0.2624, *p* = 0.0466), indicating that increased connectivity was associated with greater speech impairment severity. In addition, LFPN–AUN functional connectivity showed a significant negative correlation with MoCA scores (*r*_s_ = −0.2847, *p* = 0.0249), suggesting that increased connectivity was associated with poorer cognitive performance. These findings are consistent with emerging evidence that speech dysfunction in PD extends beyond a pure motor deficit and represents a complex clinical manifestation involving broader cognitive processes, particularly executive functions (Rohl et al. 2022).

**FIGURE 5:**
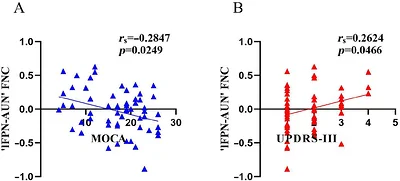
Spearman correlation analysis between LFPN‐AUN functional connectivity and MoCA and UPDRS‐III speech subscores in PD‐SD group. (A) Correlation analysis of LFPN‐AUN functional connectivity and MoCA scores; (B) correlation analysis of LFPN‐AUN functional connectivity and UPDRS‐III speech subscores. AUN, auditory network; LFPN, left frontoparietal network.

## 4 Discussion

To the best of our knowledge, this is the first study to investigate large‐scale RSN mechanisms underlying speech impairment in individuals with PD using ICA‐based rs‐fMRI. Among the seven RSNs identified, functional connectivity between the LFPN and AUN was significantly increased in PD‐SD group compared with the PD‐NON‐SD group and HCs. Notably, post hoc analyses demonstrated that this connectivity alteration was not present in the PD‐NON‐SD group relative to HCs. These findings suggest that aberrant interactions between the LFPN and AUN may represent a neural correlate associated with speech impairment in PD.

The key nodes of the left frontoparietal network and RFPN include the lateralized superior parietal lobule, inferior temporal cortex, dorsolateral prefrontal cortex, and presupplementary motor area (Boyle et al. 2024). These networks are crucial for top‐down self‐regulation, affecting cognitive, emotional, and behavioral processes (Barrós‐Loscertales et al. 2020). A recent large‐scale rs‐fMRI study has further confirmed the role of the LFPN in speech generation, establishing it as a core component of the language network (Abrams et al. 2020). The AUN primarily consists of the primary and secondary auditory cortices located in the superior temporal gyrus and transverse temporal gyrus, as well as regions such as the temporal pole and posterior insula (Beckmann et al. 2005). Sound information is initially processed in the primary auditory cortex, followed by further processing in the secondary auditory cortex. This information is then transmitted via the thalamus to higher‐order language areas, including Wernicke's area, for integration (Ćurčić‐Blake et al. 2017). Previous studies have shown that the AUN and the frontoparietal network work synergistically to regulate auditory speech perception and speech production (Braga et al. 2017). Specifically, the inferior frontal gyrus, located in the anterior part of the frontoparietal network, sends top‐down signals to the auditory cortex to coordinate its activity with speech input (Rimmele et al. 2018; Park et al. 2015). A task‐based fMRI study by Orpella et al. (2022) demonstrated a strong correlation between activation in the frontoparietal network and performance in speech‐motor synchronization tasks, underscoring the critical role of this network in integrating auditory and motor information.

The frontoparietal networks comprise distributed regions involved in executive control, cognitive regulation, and sensorimotor integration, including lateralized superior parietal and prefrontal regions. In addition, we found that enhanced LFPN–AUN connectivity was positively correlated with MDS‐UPDRS part III speech item scores and negatively correlated with MoCA scores. These findings indicate that greater LFPN–AUN connectivity is associated with more severe speech impairment and poorer cognitive performance in individuals with PD‐SD. Such alterations may reflect either compensatory recruitment or maladaptive reorganization between speech‐related motor control and higher‐order cognitive systems through the LFPN–AUN pathway (Schill et al. 2023). Consistent with this interpretation, recent studies have shown that PD‐related speech impairment is frequently accompanied by abnormal hyperactivity within cortical speech motor networks (Dai, Wang, et al. 2022). One possible explanation for this enhanced connectivity is that impaired feedforward motor control in PD may increase reliance on auditory feedback mechanisms during speech production. In response, individuals with PD may recruit additional cortical resources to compensate for disrupted auditory–motor integration, resulting in excessive auditory feedback‐related responses (Huang et al. 2019). Therefore, the observed LFPN–AUN hyperconnectivity may reflect increased engagement of top‐down cognitive resources involved in monitoring and regulating auditory–motor interactions. Collectively, these findings support a model in which disrupted information integration between the LFPN and AUN contributes to speech dysfunction in PD‐SD. From a mechanistic perspective, we propose that altered LFPN–AUN connectivity may reflect disruption of top‐down regulatory processes. Under physiological conditions, frontal LFPN regions, including the dorsolateral prefrontal cortex, contribute to the modulation of auditory processing and help prevent excessive dependence on auditory feedback during speech production (D. Liu et al. 2020). In neurodegenerative disorders, impaired prefrontal regulation has been associated with abnormal auditory feedback processing during vocal control (Ranasinghe et al. 2019). In PD, dopaminergic depletion disrupts cortico‐striatal‐frontal circuits, potentially compromising the regulatory capacity of frontal control regions (Alexander et al. 1986). Consequently, reduced efficiency of LFPN‐mediated modulation may lead to excessive engagement of AUNs. Thus, the observed hyperconnectivity may represent a maladaptive compensatory response reflecting increased but inefficient recruitment of top‐down regulatory mechanisms in PD‐related speech impairment.

Importantly, normal speech production relies on a hierarchical mechanism that must be distinguished across two temporal and cognitive levels. Early automatic processes, typically reflected by neurophysiological event‐related potentials (ERPs) within the 100–200 ms window (e.g., N1 component), represent rapid, pre‐attentive sensory processing and basic auditory‐motor feedback detection (Behroozmand et al. 2011; D. Liu et al. 2023). In contrast, later processes occurring approximately in the 500–700 ms range reflect higher‐order cognitive mechanisms, including conscious awareness, explicit self‐monitoring, and cognitive evaluation of speech output (Suchý et al. 2025). Among these, auditory feedback serves as a critical sensory signal for regulating vocal movements by enabling continuous auditory feedback processing and adjustment of self‐generated speech. When discrepancies arise between expected and perceived auditory feedback, the auditory‐vocal system dynamically adapts acoustic parameters, including fundamental frequency, intensity, and formant frequencies, to maintain speech accuracy. Research has indicated the essential role of auditory feedback in the maintenance and regulation of speech motor control (Houde and Jordan 1998). Disruption of auditory feedback regulation may impair the processing of self‐generated speech signals and compromise speech motor control through altered interactions between auditory and motor systems. Previous studies have shown that abnormalities in auditory–vocal integration, characterized by impaired auditory feedback processing and disrupted vocal motor regulation, represent important contributors to speech impairment in PD (Moreau and Pinto 2019; H. Liu et al. 2012; Sapir et al. 2007). Huang et al. (2019, 2016) demonstrated impaired auditory–vocal integration in individuals with PD and proposed that these individuals may exhibit increased dependence on auditory feedback to compensate for deficits in internal motor control. Using auditory feedback manipulation, fundamental frequency perturbation paradigms, and ERP analyses, these studies showed that external auditory cues reduced excessive reliance on auditory feedback while enhancing the P2 component of ERPs. Although P2 represents an early time‐locked electrophysiological response, accumulating ERP evidence in speech motor control indicates that P2 amplitude is modulated by higher‐order cognitive processes, including attention and working memory (Huang et al. 2019; D. Liu et al. 2020). The observed increase in P2 amplitude was associated with reduced acoustic compensation responses, suggesting that enhanced cortical processing may reflect inhibitory regulation of excessive compensatory speech behaviors. These findings imply that external auditory cues may facilitate auditory–motor integration in PD through top‐down cognitive mechanisms. In this context, the abnormal LFPN–AUN functional connectivity identified in our study provides additional evidence for disrupted auditory feedback regulation pathways in PD‐related SD, supporting the hypothesis that impaired coordination between cognitive control and auditory processing systems contributes to speech dysfunction in PD.

Although our findings provide new insights into the neural basis of speech impairment in PD, several limitations should be acknowledged. First, the cross‐sectional design prevents definitive causal interpretations. It remains unclear whether the observed LFPN‐AUN hyperconnectivity represents a primary mechanism contributing to speech dysfunction or a secondary compensatory response to impaired speech control. Second, our use of static, time‐averaged functional connectivity may have limited our ability to capture transient fluctuations in neural network organization. Given the dynamic and distributed nature of the speech control connectome (Fuertinger et al. 2015), future longitudinal studies incorporating dynamic functional connectivity (dFC) analyses will be important for characterizing abnormal temporal network states during speech production (Cao et al. 2023; Engels et al. 2018). Third, defining the SD group solely based on the MDS‐UPDRS part III speech item score represents a methodological limitation. Although this approach reflects current clinical practice, reliance on a single clinical measure with a predefined severity threshold may not fully capture the heterogeneity of speech impairment in PD. Therefore, differences in LFPN‐AUN connectivity between groups should be interpreted cautiously, as they may partially reflect variations in symptom severity rather than distinct neurophysiological phenotypes. Moreover, the limited sensitivity of this measure to subtle speech abnormalities may have resulted in the inclusion of individuals with subclinical speech alterations in the PD‐NON‐SD group. Accordingly, the observed association between LFPN–AUN connectivity and speech scores requires cautious interpretation. Future studies should incorporate comprehensive speech assessments, including objective acoustic analyses and standardized dysarthria evaluations, to achieve more precise phenotypic characterization. Fourth, although the unequal sample sizes between the PD‐SD and PD‐NON‐SD groups reflect the reported prevalence distribution of SD in PD, the relatively small PD‐NON‐SD subgroup may reduce statistical robustness. Nevertheless, post hoc power analysis indicated that the current sample size provided adequate power (85%) to detect the large effect size observed in the primary FNC analysis (Cohen's *d* = 0.85). In addition, the slightly higher proportion of females in the PD cohort likely reflects consecutive convenience sampling rather than population‐level sex distribution. The absence of stratification by motor phenotype (e.g., tremor‐dominant vs. postural instability/gait difficulty subtypes) represents another limitation, as distinct motor phenotypes may influence functional connectivity patterns (Helmich et al. 2012). Future multicenter studies with larger and balanced cohorts will be necessary to validate and extend these findings. Finally, the correlational nature of our clinical analyses limits the ability to determine causal relationships among motor dysfunction, cognitive decline, and impaired sensorimotor integration in the development of SD. Future research could employ a combination of functional neuroimaging, electrophysiological mapping techniques, and acoustic analysis to more deeply explore these mechanisms. Clinically, this hyperconnected LFPN‐AUN pathway may serve as a specific neuroanatomical target. Future studies should determine whether targeted neuromodulation of this network interaction can restore auditory–motor balance and improve speech outcomes in individuals with PD.

## 5 Conclusion

This study identifies increased functional connectivity between the LFPN and the AUN in the PD‐SD group. These findings suggest that altered integration among large‐scale RSNs involved in motor planning, cognitive control, and auditory processing may contribute to speech impairment in PD.

## Author Contributions

**Shan Deng**: conceptualization, methodology, writing – original draft. **Yongqing Xiao**: data curation, formal analysis, writing – original draft. **Zimei Dong**: funding acquisition, investigation, resources, validation, visualization. **Cai Zhong**: funding acquisition, investigation, resources, validation, visualization. **Yu Yu**: funding acquisition, investigation, resources, validation, visualization. **Chunxiao Yang**: funding acquisition, investigation, resources, validation, visualization. **Liya Pan**: funding acquisition, investigation, resources, validation, visualization. **Baohui Weng**: funding acquisition, investigation, resources, validation, visualization. **Yuan Chen**: funding acquisition, investigation, resources, validation, visualization. **Ziming Ye**: funding acquisition, investigation, resources, validation, visualization. **Ying Liu**: supervision, writing – review and editing. **Chao Qin**: conceptualization, project administration, software, supervision, writing – review and editing.

## Funding

This study was supported by the Guangxi Natural Science Foundation (Grant No. 2025GXNSFAA069411), the Liuzhou Science and Technology Plan Project (Grant No. 2024YB0103A009), and the project supported by Liuzhou Committee of Science and Technology (Grant No. 2024YB0103B016), and the Self‐Funded Research Project of the Health Commission of Guangxi Zhuang Autonomous Region (Grant Nos. Z‐B20241493 and Z‐B20241495).

## Ethics Statement

All procedures performed in this study involving human participants were in accordance with the ethical standards of the institutional and/or national research committee and with the 1964 Helsinki Declaration and its later amendments or comparable ethical standards. The study was approved by the Ethics Committee of the Liuzhou Workers’ Hospital.

## Consent

Informed consent was obtained from all individual participants included in the study.

## Conflicts of Interest

The authors declare no conflicts of interest.

## Data Availability

The datasets generated and analyzed during the current study are available from the corresponding author on reasonable request.
